# Supplementary material for: Disrupting butterfly caterpillar microbiomes does not impact their survival and development
Source: Proc Biol Sci. 2019 Dec 18;286(1917):20192438. doi: 10.1098/rspb.2019.2438 (PMC6939933; doi:10.1098/rspb.2019.2438)
Supplement: Supplementary figures, methods and tables [file rspb20192438supp1.pdf]

SUPPLEMENTARY INFORMATION

Disrupting butterfly caterpillar microbiomes does not impact their survival and development

Kruttika Phalnikar, Krushnamegh Kunte and *Deepa Agashe

National Centre for Biological Sciences, GKVK Campus, Bellary road, Bangalore, India

*Correspondence: dagashe@ncbs.res.in

Proceedings of the Royal Society B: Biological Sciences

DOI: 10.1098/rspb.2019.2438

SUPPLEMENTARY FIGURES

Figure S1: Life stages and larval host plants of *Danaus chrysippus* and *Ariadne merione*. Images are sourced from the Butterflies of India website (http://www.ifoundbutterflies.org): ***Danaus chrysippus*:** photographer: egg: Tushar Bhagwat, caterpillar, adult and host plant: Krushnamegh Kunte, pupa: Dr. Saji K. ***Ariadne merione*:** photographer: egg, caterpillar, adult and host plant: Krushnamegh Kunte, pupa: Hemant Ogale, (used with permission).

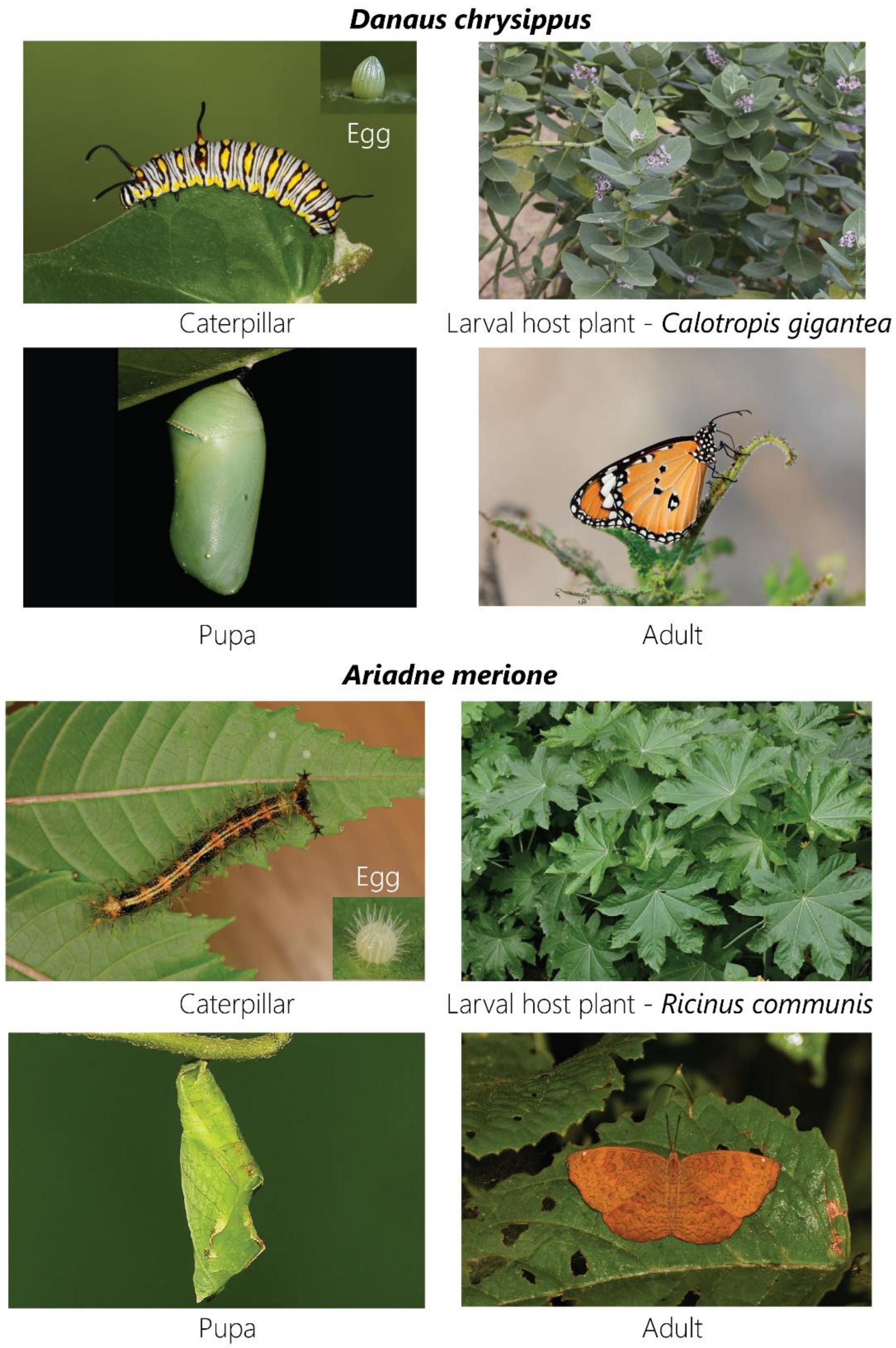

Figure S2: Effect of antibiotic treatment and dietary sterilization on bacterial communities of *D. chrysippus* and *A. merione* larvae. Panels (A1-A3): Stacked bar plots show the relative abundance of the five most abundant bacterial taxa (OTUs, identified to the lowest taxonomic level possible) in each treatment group, n=2-3 larvae per treatment. For microbiome analysis, we used *D. chrysippus* larvae from block 2 (dietary sterilization) and block 3 (antibiotic treatment), and *A. merione* larvae from block 1 (antibiotic treatment) (see SI tables S5-S7 and figures S7-S15).

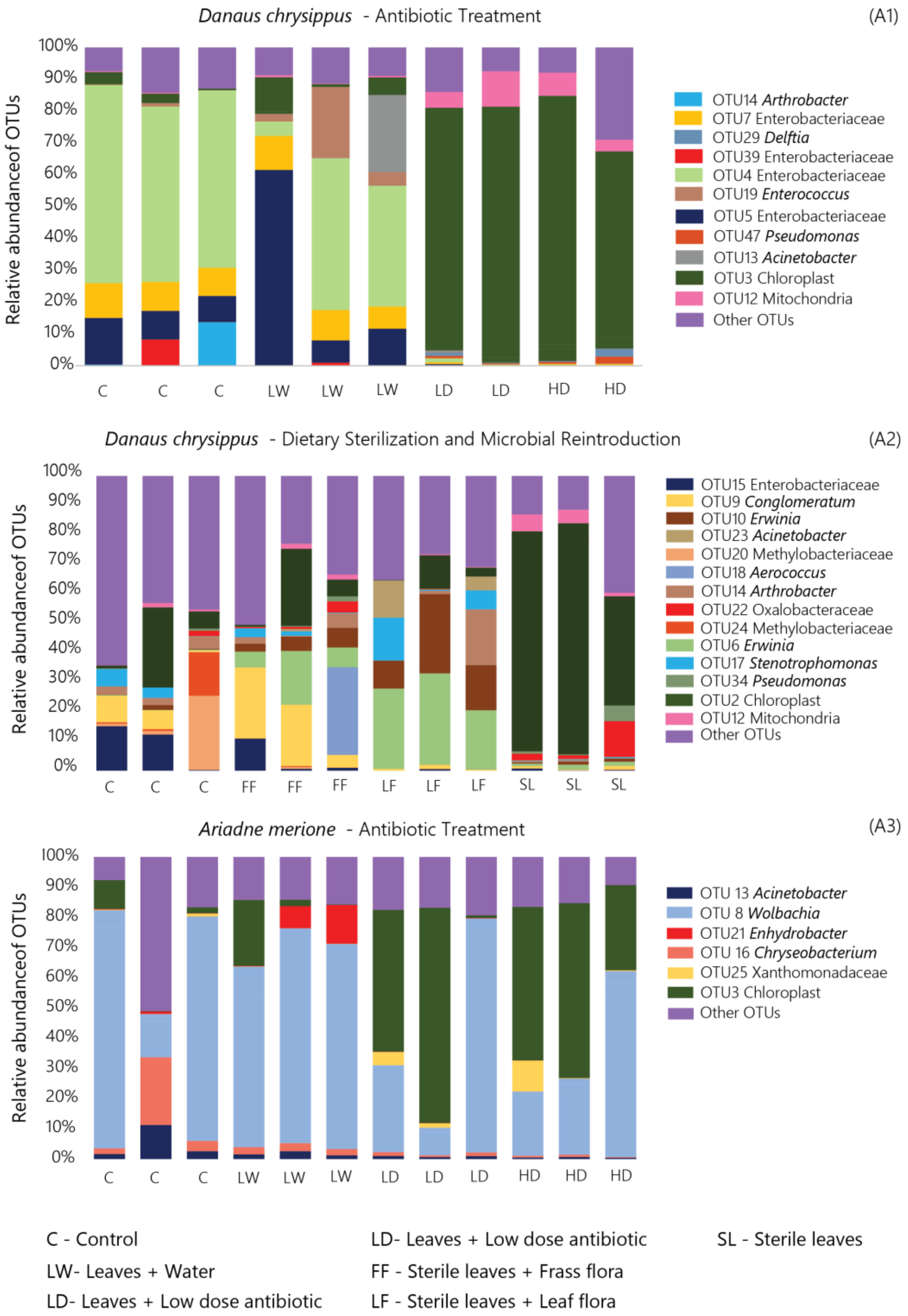

Figure S3: Variation in bacterial communities of larvae across treatments. Panels show (unconstrained) principle component analysis (PCA) of bacterial communities across experimental treatments. The figure represents the same analysis shown in figure 2; however, here we colour each of treatment separately, which are combined in figure 2. Axes represent principle components (PC) explaining maximum variation in the data. Values in parentheses show the proportion of variation explained by each PC. Dietary sterilization and antibiotic administration both significantly affected bacterial communities (Permutational multivariate ANOVA, p<0.05, 10,000 permutations).

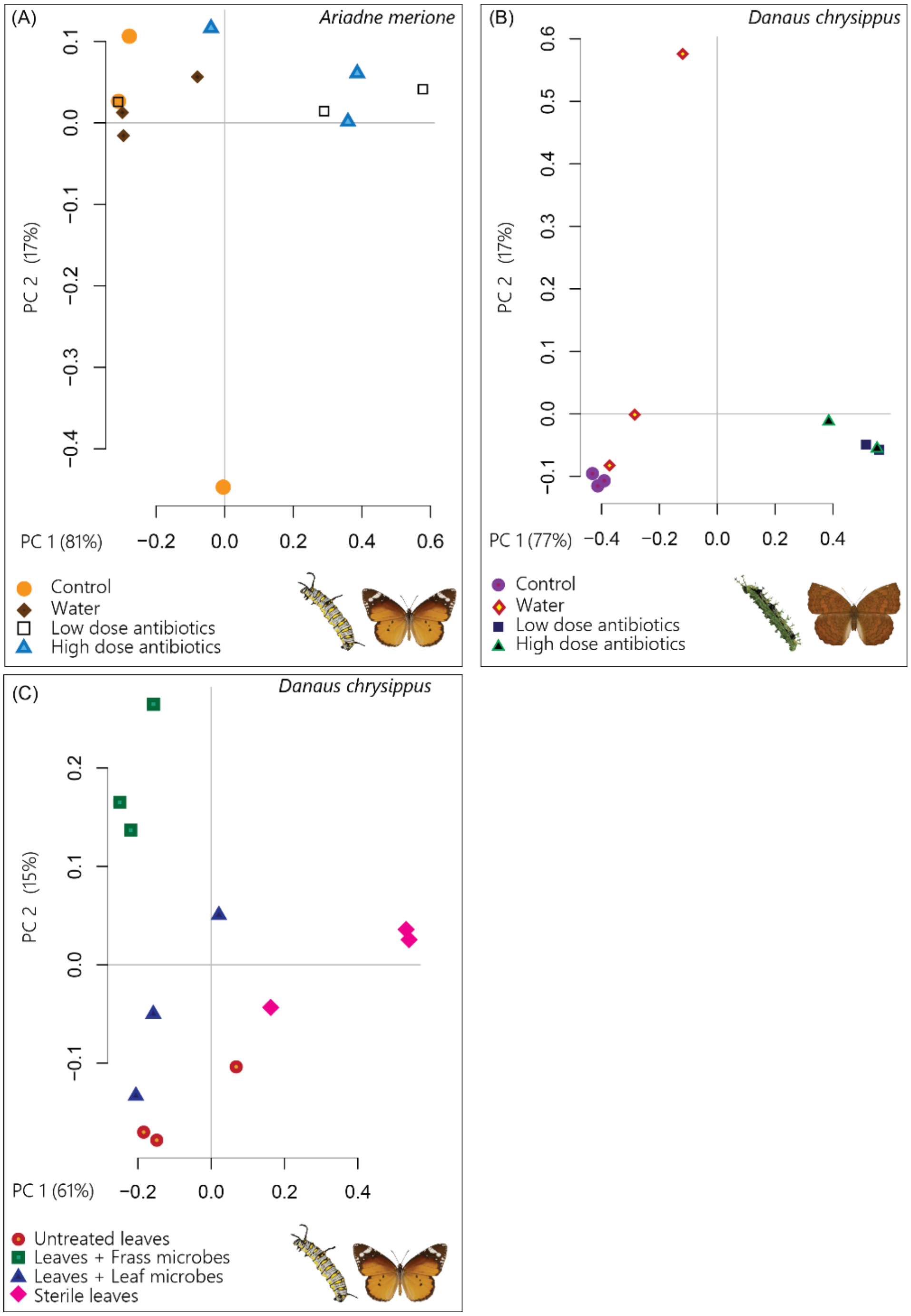

Figure S4: Variation in larval bacterial communities across treatments (CAPdiscrim analysis). Panels show Constrained Analysis of Principal Coordinates (CAP) of communities based on the composition and relative abundance of bacterial OTUs in control and treated groups. Axis labels indicate the proportion of between-group variance (%) explained by the first two linear discriminants (LD1 and LD2). In all panels, LD1 explains 100% between-group variance. Ellipses represent 95% confidence intervals. For each panel, we observed a significant variation in bacterial communities of treated and control group larvae (multivariate ANOVA, panel A, $p = 2.024e^{-09}$, panel B, $p = 0.00056$, panel C, $p = 0.016$). Larvae with intact and perturbed gut bacterial communities represent control and treated groups, respectively.

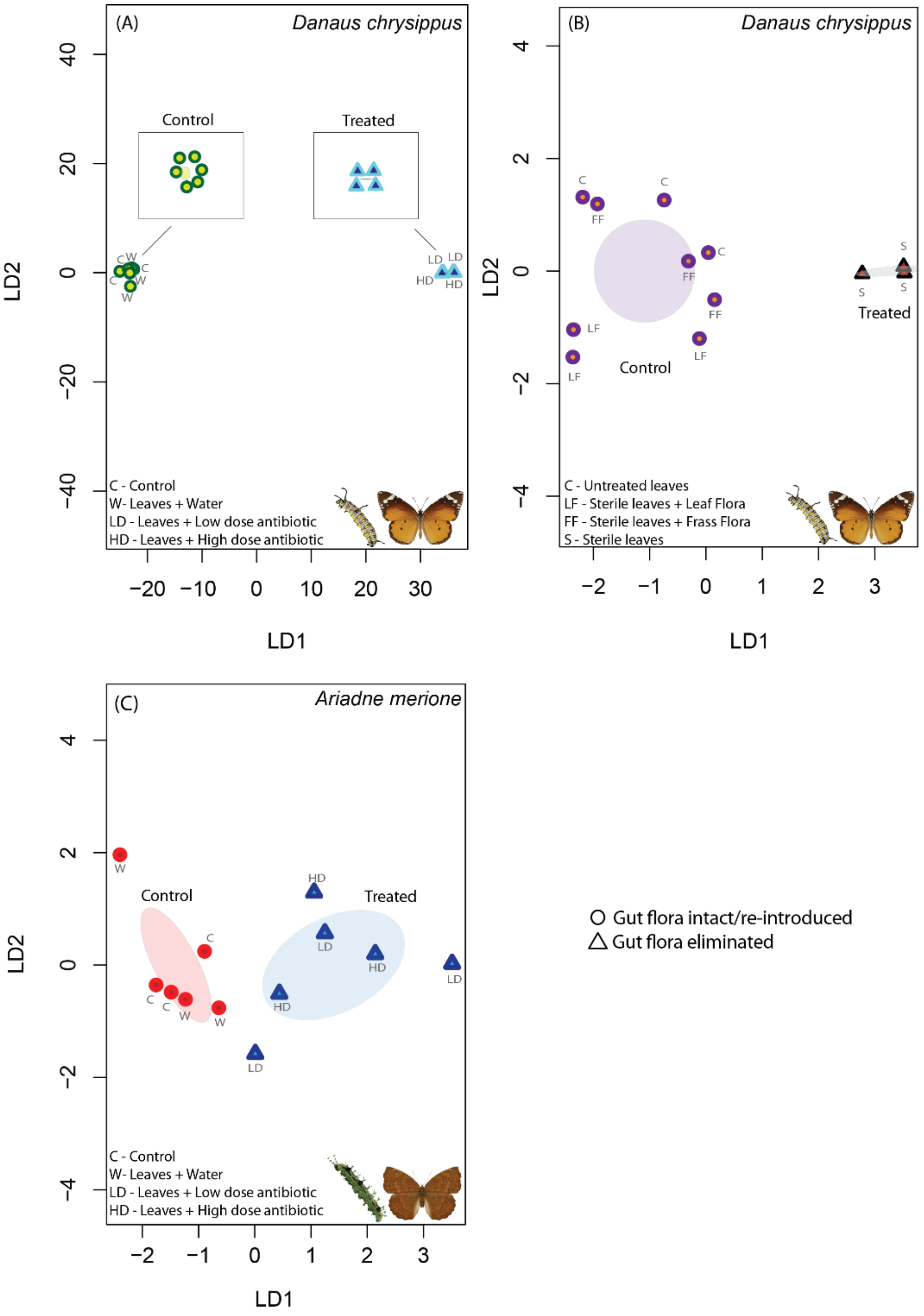

Figure S5: Variation in larval bacterial load across treatments. Barplots show the magnitude of amplification of the bacterial 16S rRNA gene relative to the host 18S rRNA gene (internal control), calculated as $2^{-\Delta CT}$, where ΔCT (cycle threshold) = $CT_{target} - CT_{internal\ control}$ CT (n=2-3 larvae). Error bars represent standard deviation. Values in parentheses indicate the mean fold reduction in bacterial abundance in treated samples as compared to control samples, calculated as ($2^{-\Delta CT}{}_{control}$ / $2^{-\Delta CT}{}_{treated}$).

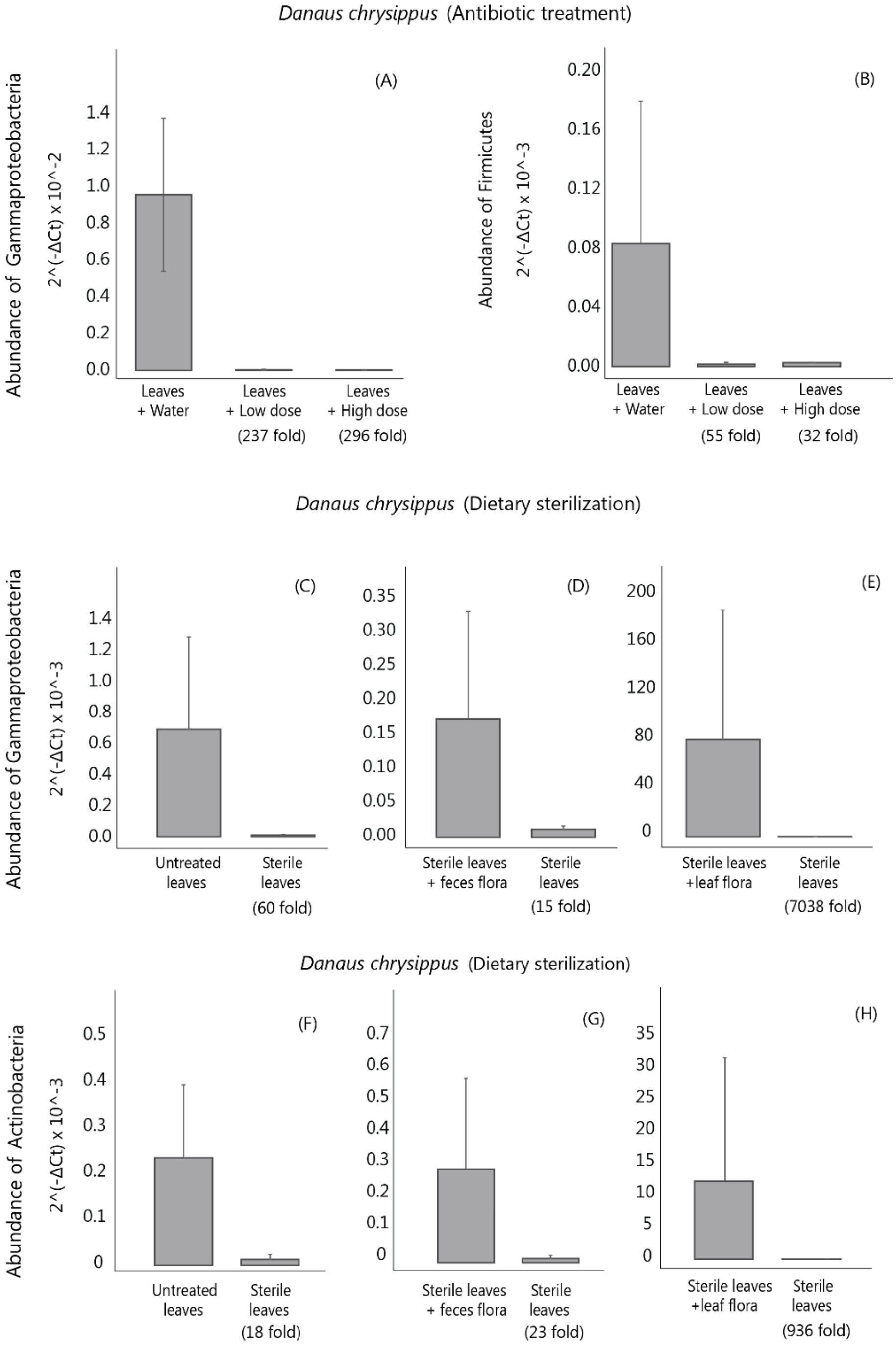

Figure S6: Boxplots (median with quartiles; whiskers show data range) show the relative abundance of reads assigned to chloroplasts and mitochondria (combined) across control and treated groups.

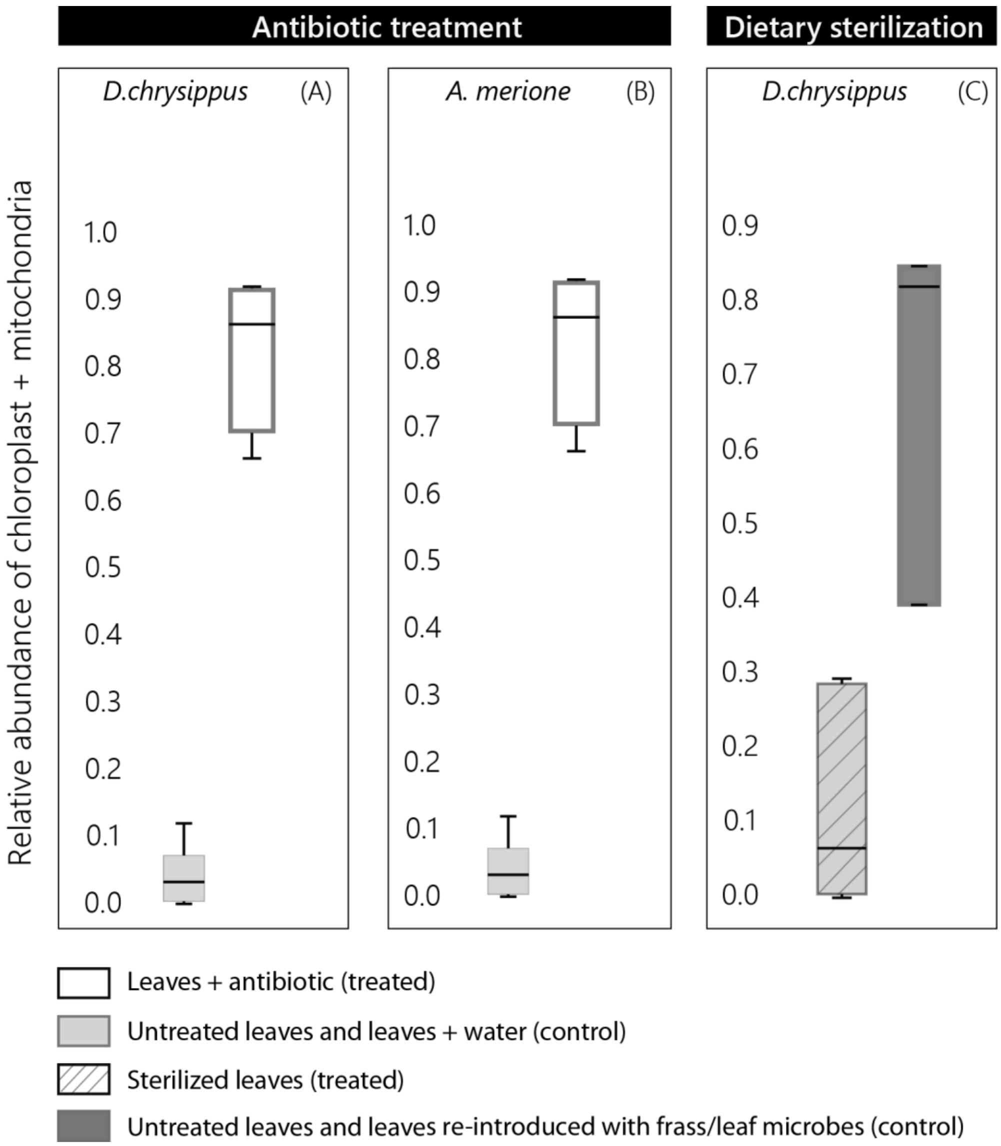

Figure S7: Effect of antibiotic administration on fitness-related traits of *D. chrysippus*. Panels show different fitness measures for experimental block 1. We did not observe a significant treatment effect for any measurement. For each treatment group (GLM, Tukey's post hoc test for multiple comparisons, p > 0.05), n=5-8 individuals. Fitness measurements for other blocks are shown in figures S8-S10 and results for all 4 blocks are summarized in table S5. The exact number of individuals included in each treatment for each fitness measurement is shown in table S9.

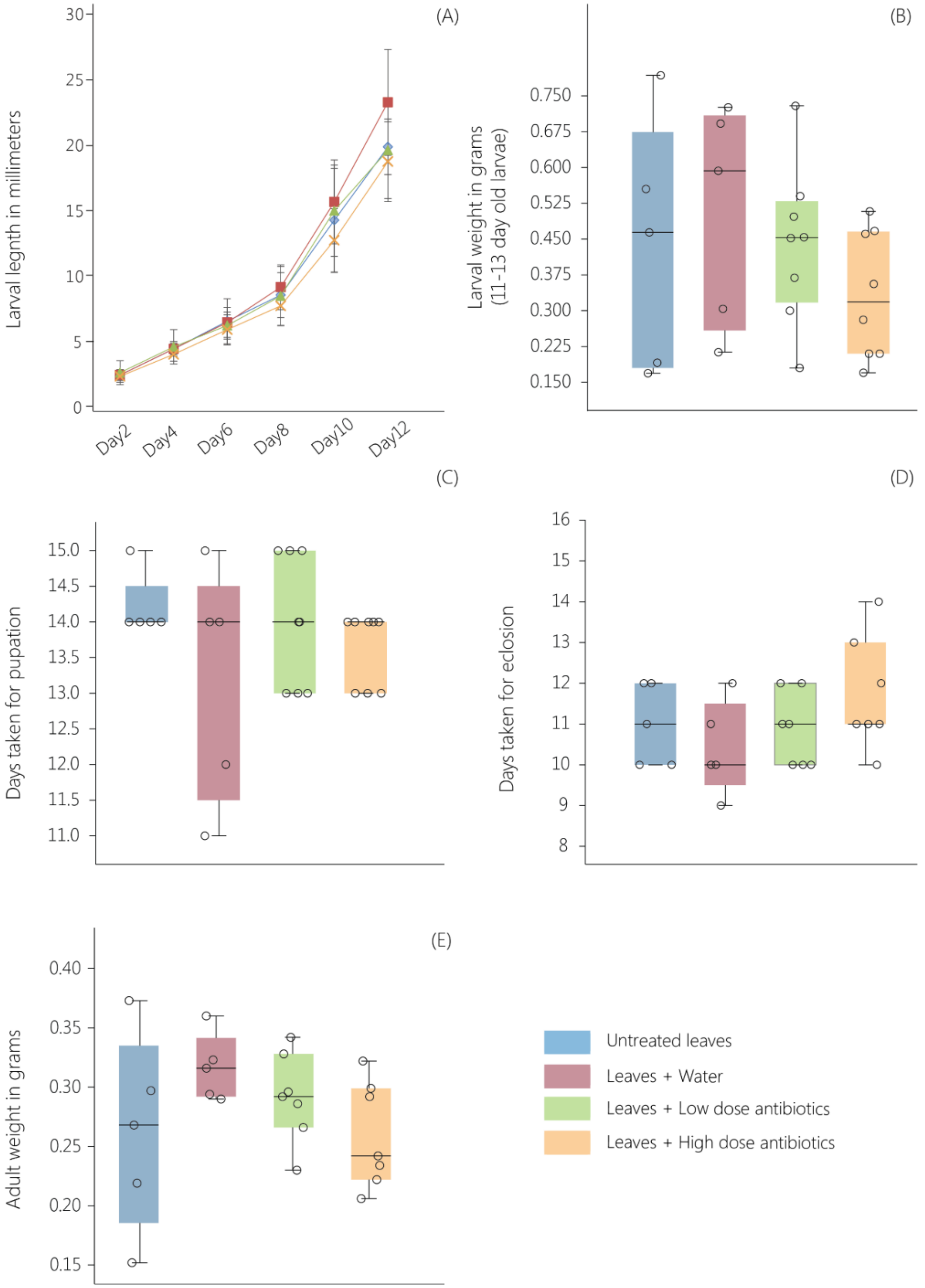

Figure S8: Effect of antibiotic administration on fitness-related traits of *D. chrysippus*. Panels show different fitness measures for experimental block 2. Asterisks indicate a significant difference between treatment groups (GLM, Tukey's test for multiple comparisons, $p < 0.05$). For each treatment group, n= 4-18 individuals. Fitness measurements for other blocks are shown in figures S7, S9 and S10, and results for all 4 blocks are summarized in table S5. The exact number of individuals included in each treatment for each fitness measurement is shown in table S9.

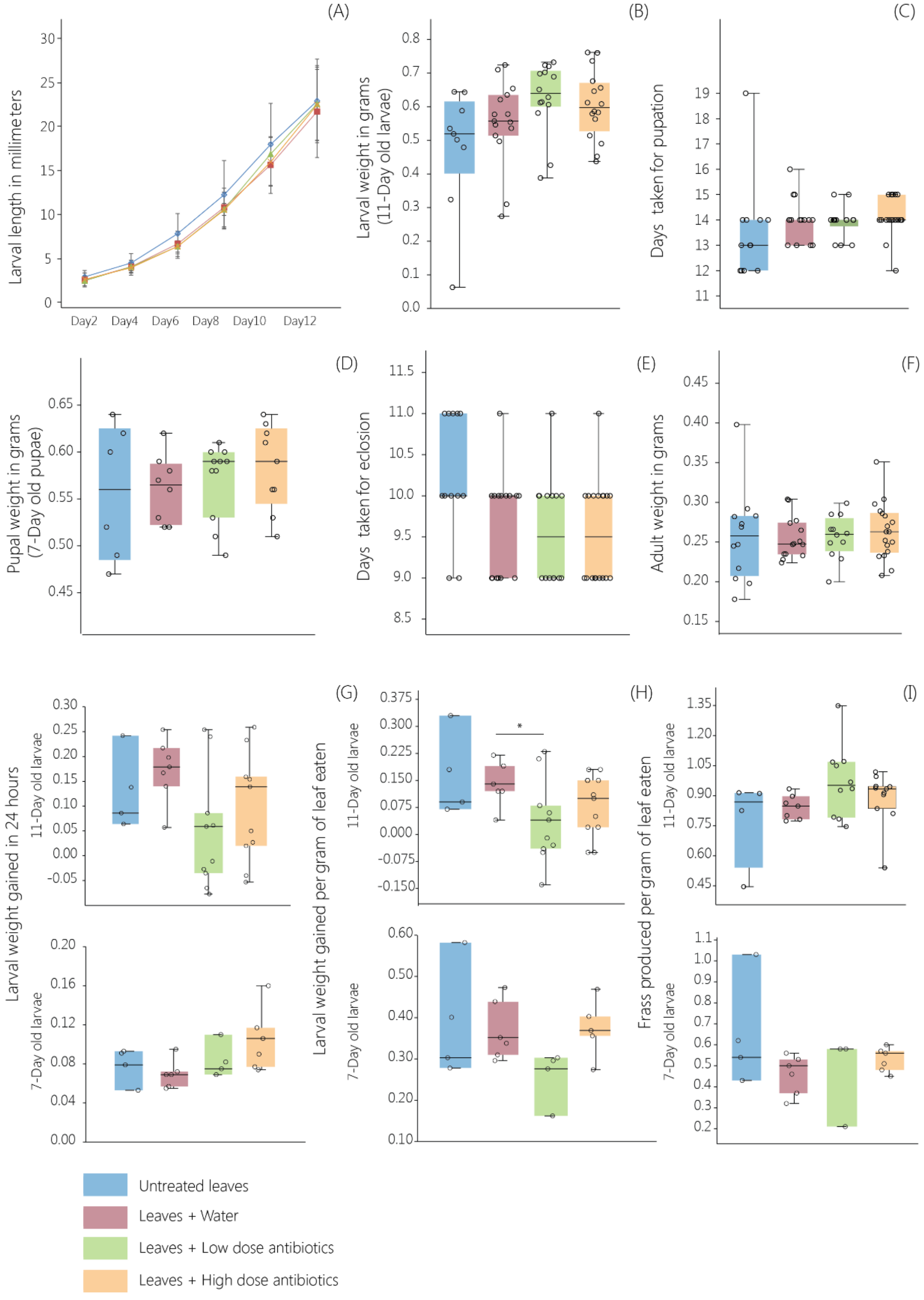

Figure S9: Effect of antibiotic administration on fitness-related traits of *D. chrysippus*. Panels show different fitness measures for experimental block 3. We did not observe a significant treatment effect for any measurement (GLM, model: fitness ~ treatment, Tukey's post hoc test for multiple comparisons, p>0.05). For each treatment group, n = 9-25 individuals. Fitness measurements for other blocks are shown in figures S7, S8 and S10, and results for all 4 blocks are summarized in table S5. The exact number of individuals included in each treatment for each fitness measurement is shown in table S9.

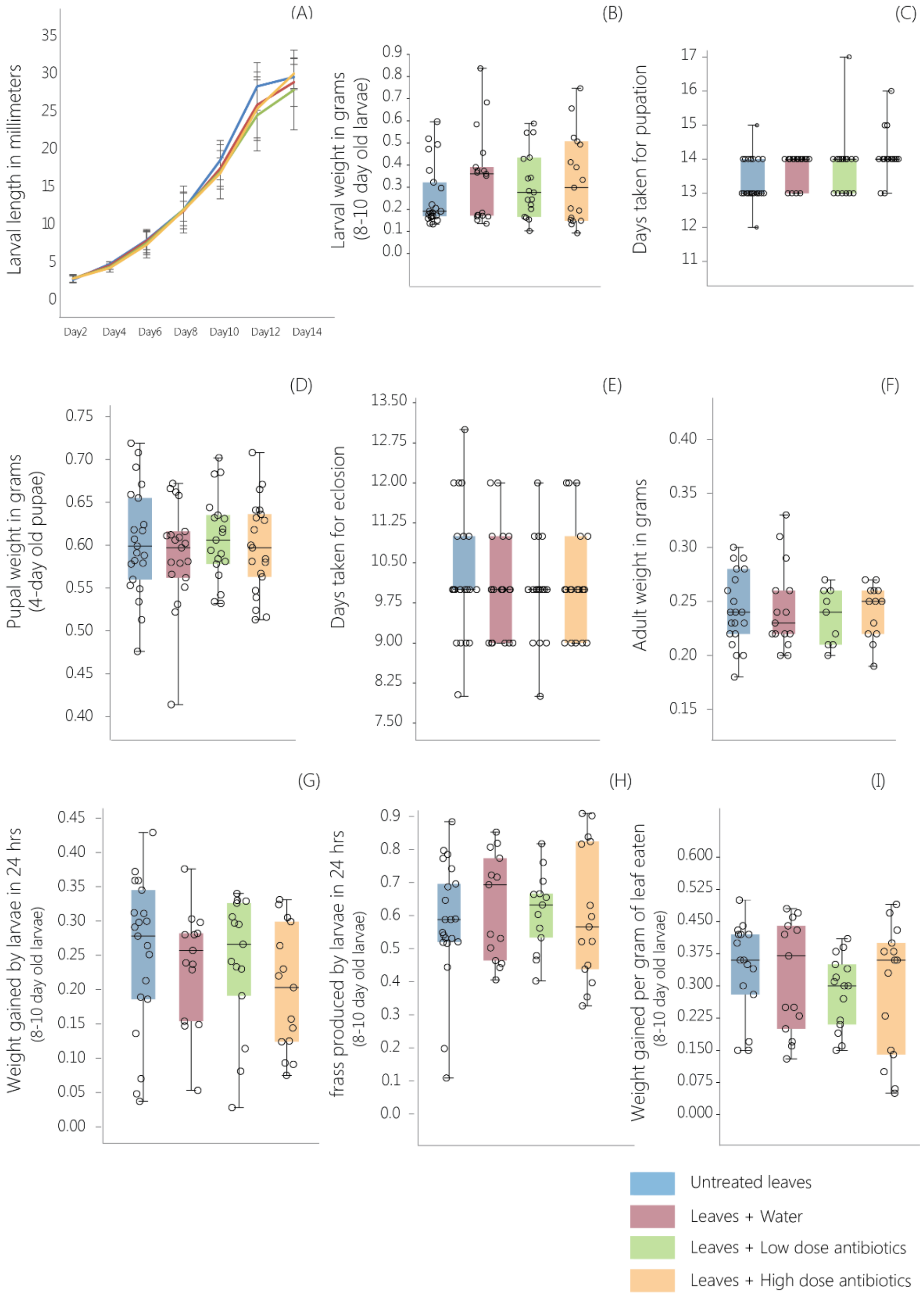

Figure S10: Effect of antibiotic administration on fitness-related traits of *D. chrysippus*. Panels show different fitness measures for experimental block 4. No treatment effects for any measurement are significant. For each treatment group, n= 4-13 individuals. Fitness measurements for other blocks are shown in figures S7-S9 and results for all 4 blocks are summarized in table S5. The exact number of individuals included in each treatment for each fitness measurement is shown in table S9.

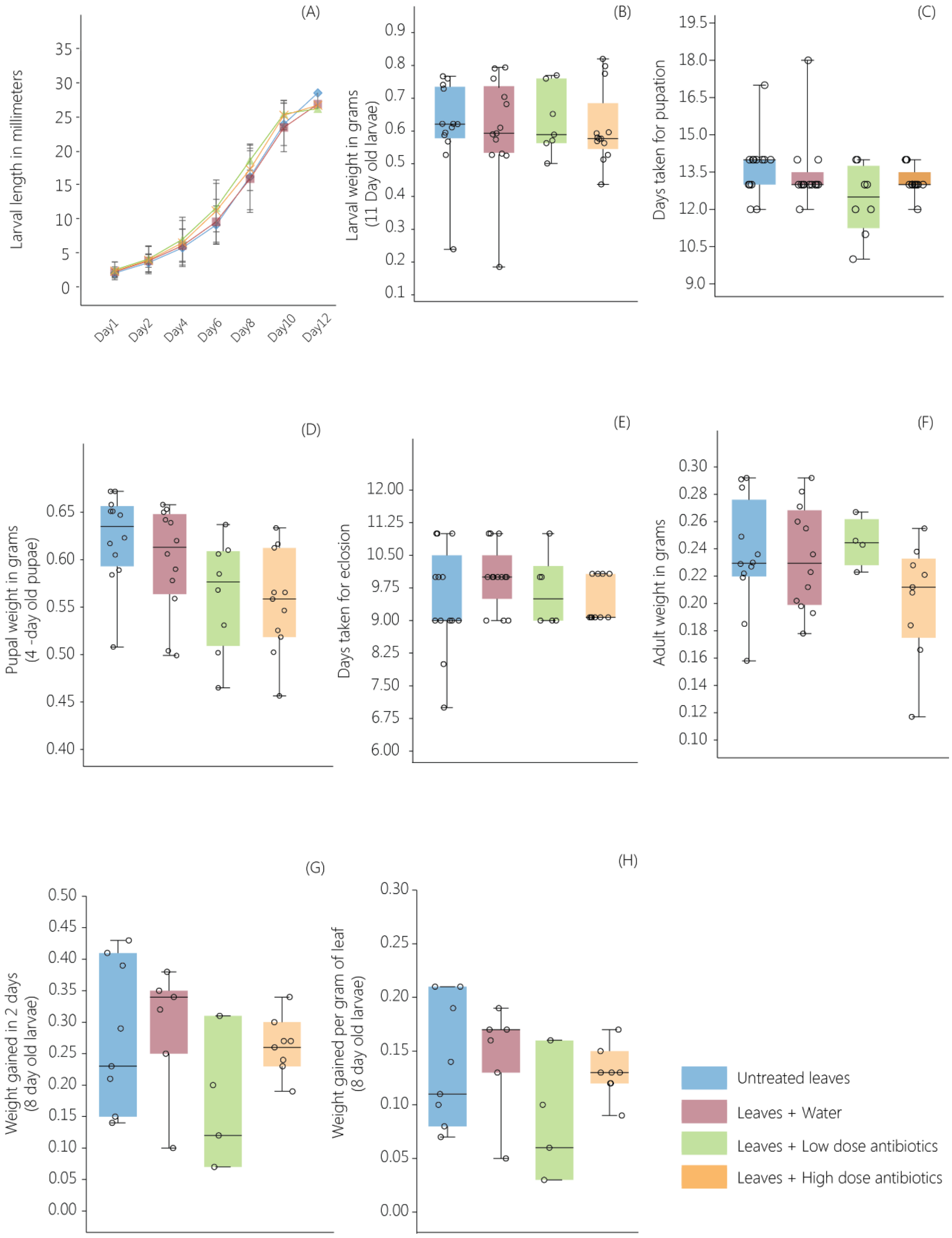

Figure S11: Effect of antibiotic administration on fitness-related traits of *A. merione*. Panels show different fitness measures for experimental block 1. We did not observe a significant treatment effect for any measurement (GLM, model: fitness ~ treatment, Tukey's post hoc test for multiple comparisons, $p>0.05$). For each treatment group, n= 9-16 individuals. Fitness measurements for other blocks are shown in figure S12 and results for all 3 blocks are summarized in table S6. The exact number of individuals included in each treatment for each fitness measurement is shown in table S9.

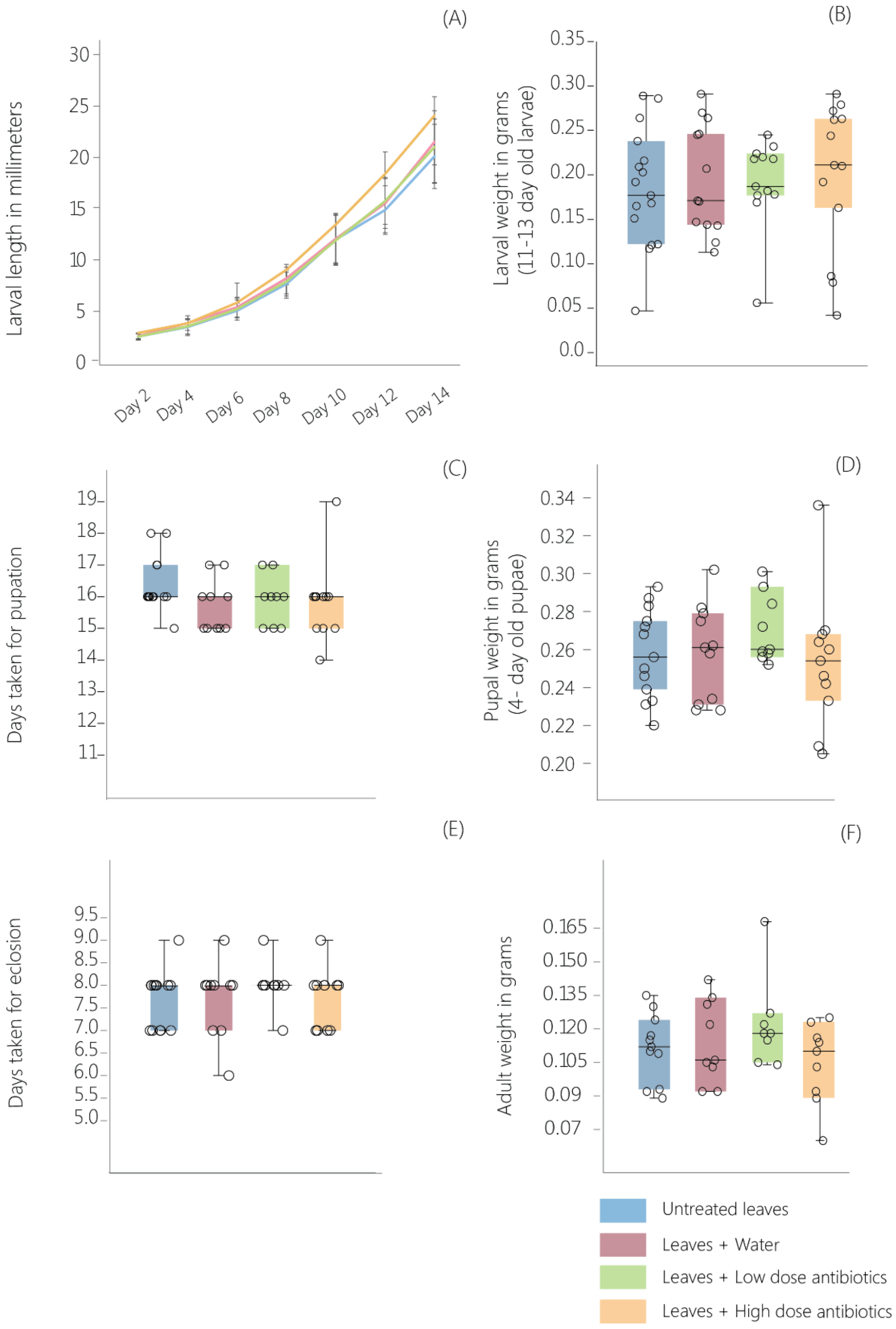

Figure S12: Effect of antibiotic administration on fitness-related traits of *A. merione*. Panels show different fitness measures for experimental blocks 2 and 3. Asterisks indicate a significant difference between treatment groups (GLM, Tukey's test for multiple comparisons, $p < 0.05$). n= 5-12 individuals per treatment for block 2 and n=3-8 for block 3. Fitness measurements for another block is shown in figures S11 and results for all 3 blocks are summarized in table S6. The exact number of individuals included in each treatment for each fitness measurement is shown in table S9.

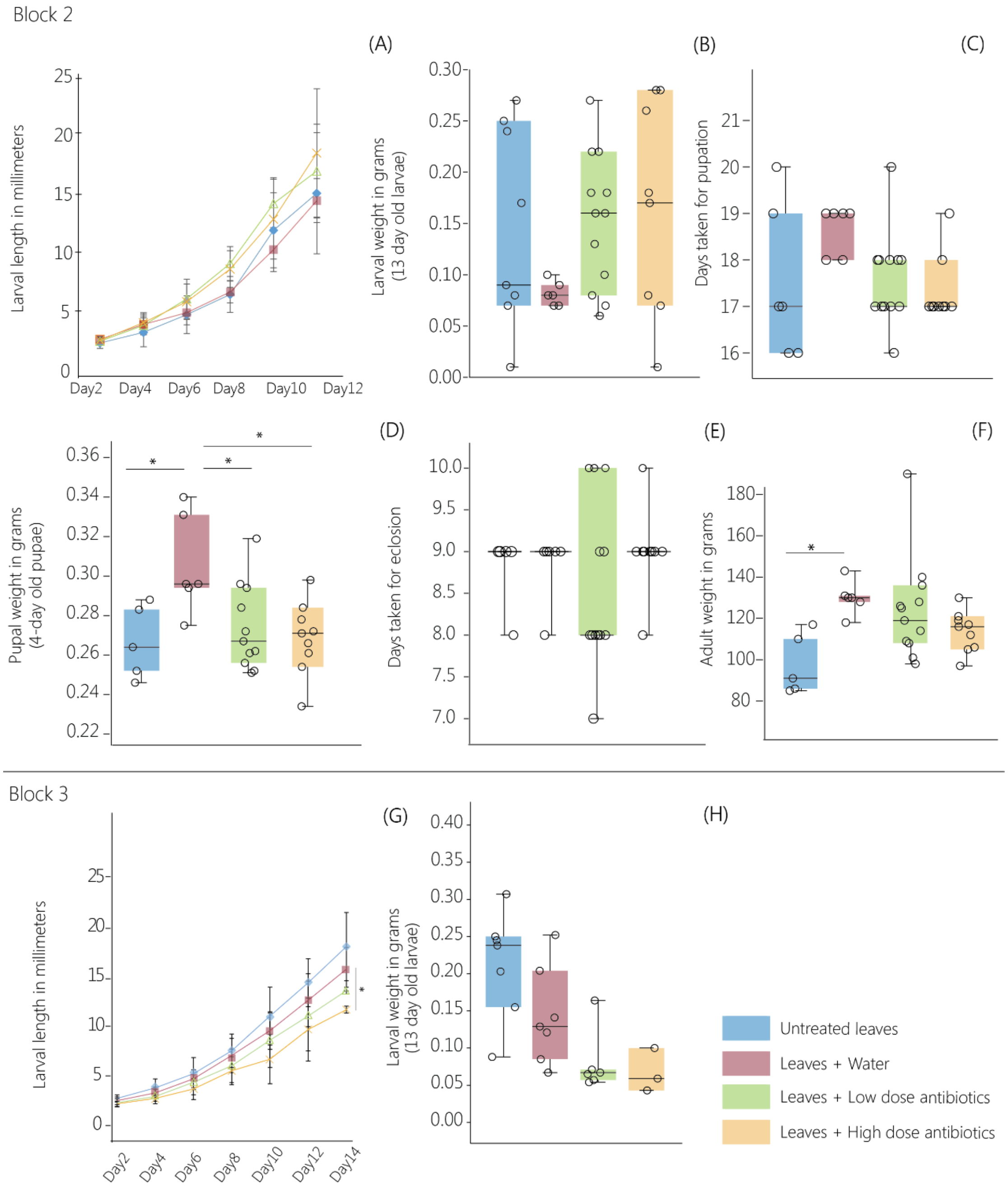

Figure S13: Effect of dietary sterilization on fitness-related traits of *D. chrysippus*. Panels show different fitness measurements for experimental block 1. Asterisks indicate a significant difference between control and treatment groups (GLM, model: fitness ~ treatment, Tukey's post hoc test for multiple comparisons, $p < 0.05$). For each treatment group, n=~8 individuals. Fitness measurements for other blocks are shown in figures S14 and S15 and results for all 3 blocks are summarized in table S7. The exact number of individuals included in each treatment for each fitness measurement is shown in table S9.

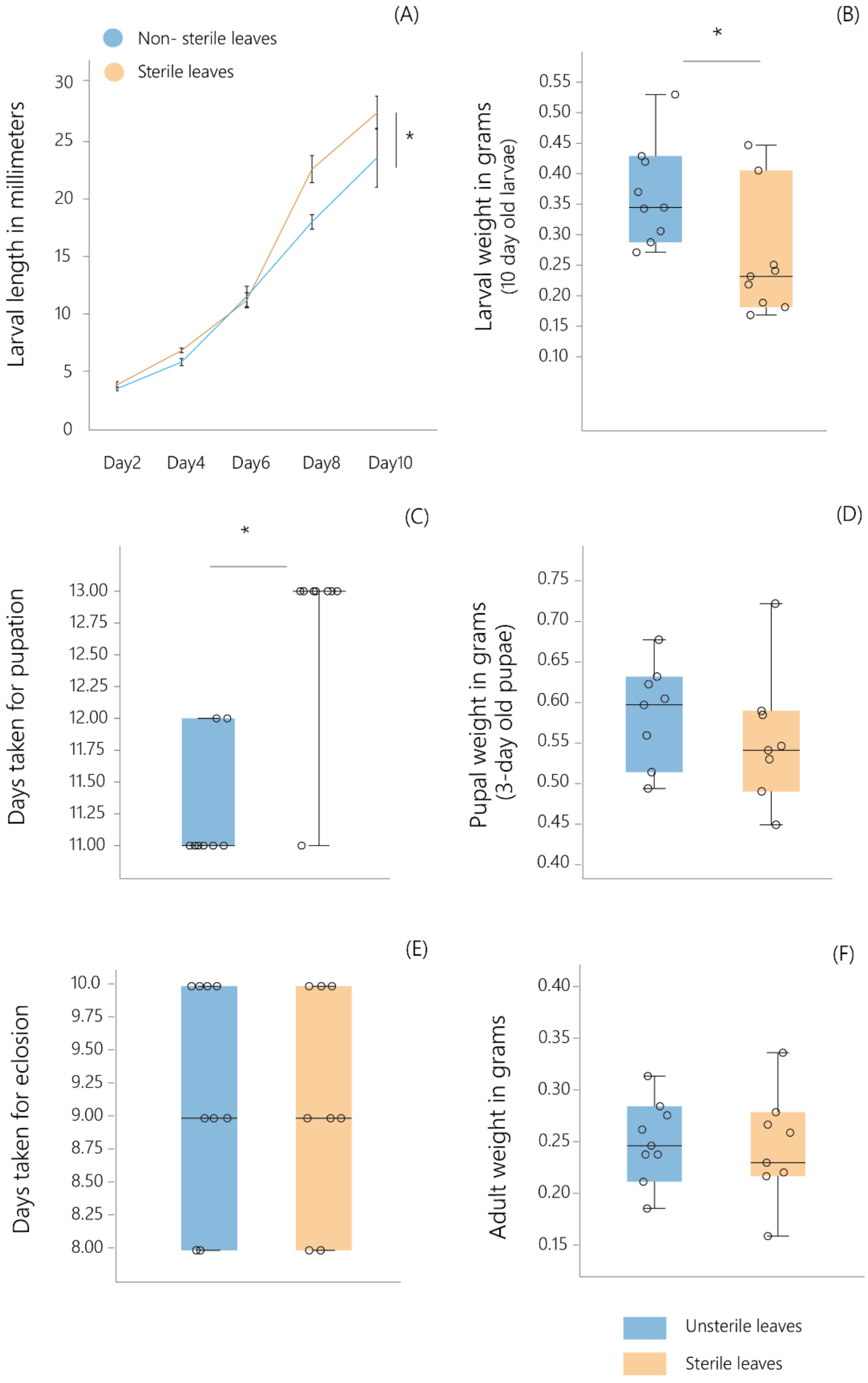

Figure S14: Effect of dietary sterilization and microbial re-introduction on fitness-related traits of *D. chrysippus*. Panels show different fitness measures for experimental block 2. Asterisks indicate a significant difference between treatment groups (GLM, Tukey's test for multiple comparisons, $p < 0.05$). For each treatment group, n= 6-13 individuals. Fitness measurements for other blocks are shown in figures S13 and S15, and results for all 3 blocks are summarized in table S7. The exact number of individuals included in each treatment for each fitness measurement is shown in table S9.

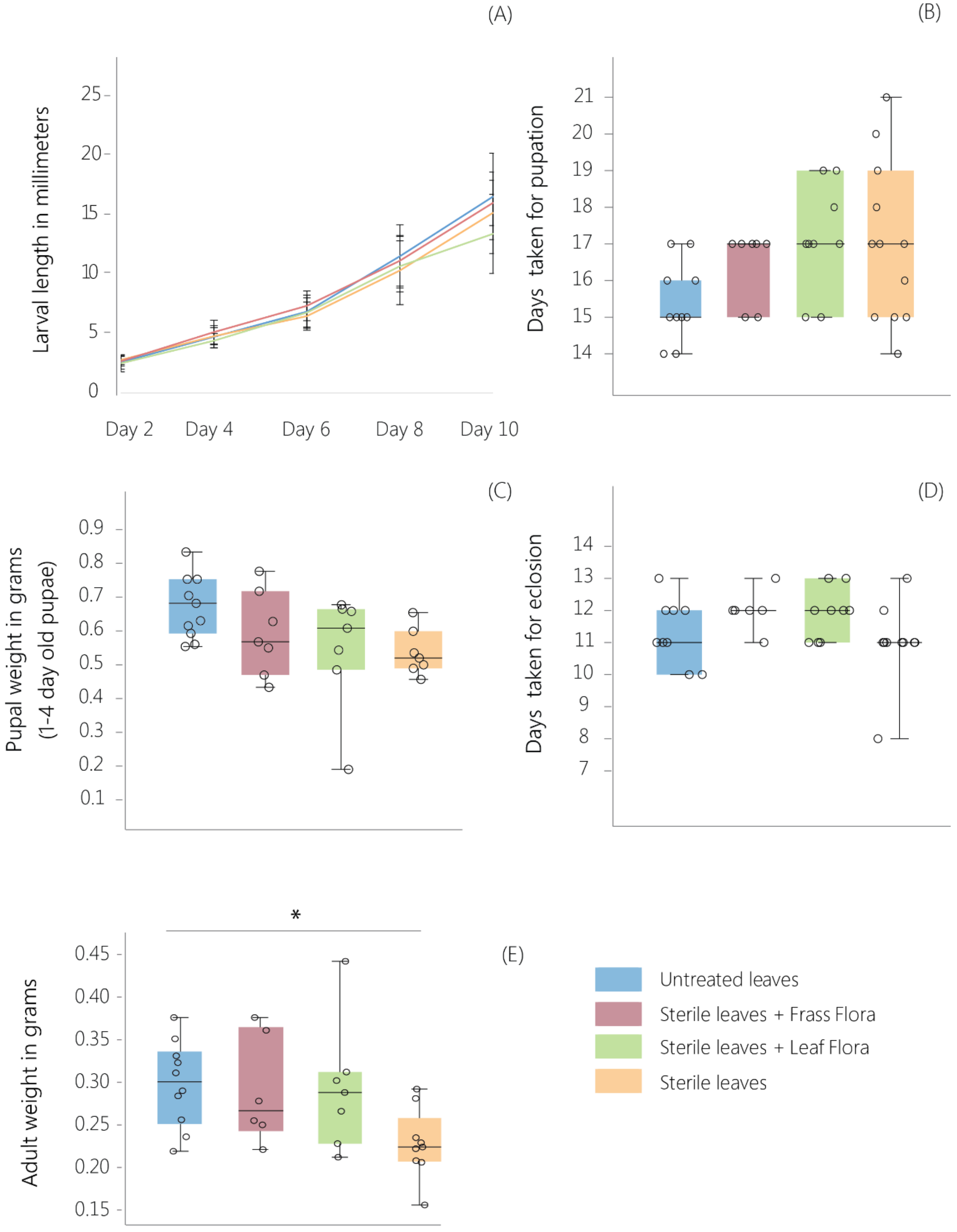

Figure S15: Effect of dietary sterilization and microbial re-introduction on fitness-related traits of *D. chrysippus*. Panels show different fitness measures for experimental block 3. Asterisks indicate a significant difference between treatment groups (GLM, Tukey's post hoc test for multiple comparisons, $p < 0.05$). For each treatment group, n= 6-8 individuals. Fitness measurements for other blocks are shown in figures S13 and S14, and results for all 3 blocks are summarized in table S7. The exact number of individuals included in each treatment for each fitness measurement is shown in table S9.

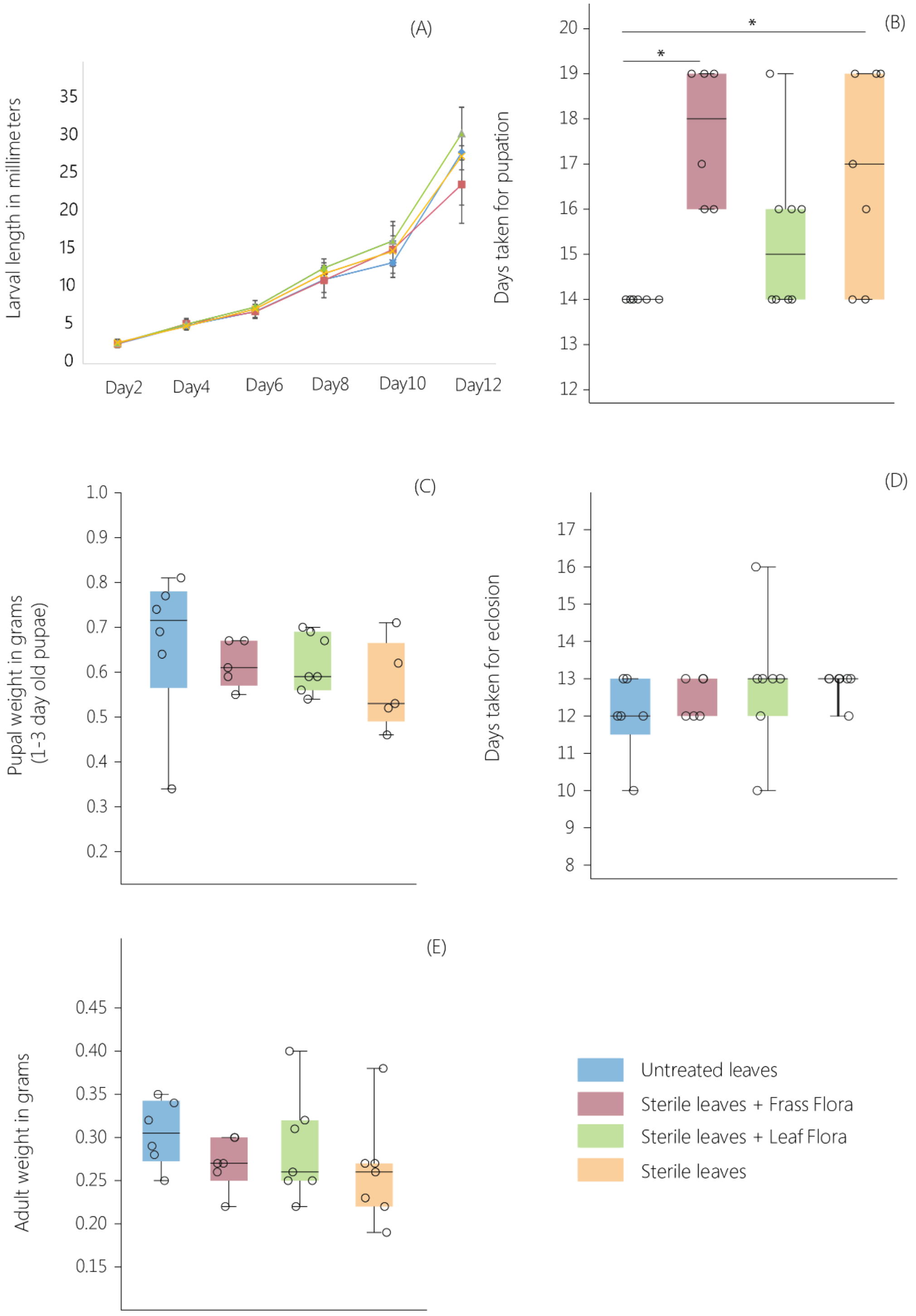

SUPPLEMENTARY METHODS

Antibiotic treatment: We selected antibiotic concentrations based on previous studies [1–3] with other insects that reported a significant reduction in gut bacteria. For *D. chrysippus*, in two out of four experimental blocks, we painted the antibiotic solution on the leaves using a sterile paintbrush; for the other two blocks, we sprayed the antibiotic solution on leaves. For *A. merione*, we sprayed the antibiotic solution on leaves in all blocks. Each spray delivered ~150-200 µl antibiotic solution; we sprayed each side of each leaf 4-6 times.

Determining larval bacterial communities: To quantify the degree of disturbance in bacterial communities of larvae fed with antibiotics and sterile diet, we sequenced the bacterial 16S rRNA gene on an Illumina MiSeq platform, at our in-house sequencing facility. We extracted DNA from whole larvae from control and treated groups (n=2-3) using a Wizard genomic DNA extraction kit (Promega). Before DNA extraction, we surface-sterilized larvae using 70% ethanol and 10% bleach, followed by 3 washes with sterile water, in order to remove surface contaminants. As shown in an earlier report [4], DNA extraction kits can also introduce bacterial contamination. Hence, we tested our kit for contaminants, performing one mock DNA extraction (without any animal tissue) as a negative control. We performed 2 rounds of PCR as part of the library preparation protocol and quantified the amount of amplified PCR product using Qubit, a sensitive method for DNA quantification. For all larval samples, we obtained ~80 ng/µl DNA per sample; however, we could not detect any amplification from the negative control [5]. This suggested that the probability of bacterial contamination from our DNA extraction kits was very low.
After extracting DNA from larvae, we amplified the V3-V4 hypervariable region of the 16S rRNA gene using 300 bp paired-end sequencing as per the standard Illumina MiSeq protocol [6]. We analyzed de-multiplexed sequences using QIIME (version 1.9.1) [7]. We filtered reads for quality using a minimum quality score of q30 and removed chimeric sequences using USEARCH (version 6.1) [8]. We assembled filtered reads into Operational Taxonomic Units (OTUs) with 97% sequence similarity using UCLUST, with the 'open-reference OTU picking' method in QIIME. To determine taxonomy, we compared one representative sequence from each OTU against the Green Genes 16S ribosomal gene database (Greengenes Database Consortium, version gg_13_5) using default QIIME parameters. We used Permutational multivariate ANOVA (permanova, Adonis, package "Vegan") [9] in R [10] to compare bacterial communities of treated and untreated larvae.
For obtaining bacterial OTUs, we used the following commands in QIIME:

- *multiple_join_paired_ends.py* (Join forward and reverse reads)
- *multiple_split_libraries_fastq.py* (q score >29) (Filter low quality reads)
- *identify_chimeric_seqs.py* and *filter_fasta.py* (Identify and filter chimeric sequences)
- *pick_open_reference_otus.py* (Pick bacterial OTUs)

For representing the dominant bacterial community members of *D. chrysippus* and *A.merione* larvae, we selected the five most abundant bacterial OTUs as described earlier [5]. We used both constrained and unconstrained ordination analysis to visualize the differences in bacterial communities of treated and control samples. Unconstrained ordination analyzes samples without any *a priori* information about groups (e.g. control vs. treated), whereas in constrained ordination, sample groups are pre-defined. We performed Principle Component Analysis as unconstrained ordination using the package "pca3d" [11] in R. As constrained ordination, we performed Canonical Analysis of Principal Coordinates based on discriminant analysis (CAPdiscrim) using the R package "BiodiversityR" [12].

Quantifying bacterial abundance using quantitative PCR (qPCR): We set up a 10µl PCR reaction for each sample using 5µl SYBR green (Maxima SYBR Green/ROX qPCR Master Mix (2X), Thermo Fischer Scientific), 1µl forward and reverse primer each (10µM), 1 µl larval DNA extract (~200ng DNA) and 2µl water. We set up qPCR reactions in 384 well plates (MicroAmp™ Optical 384-Well Reaction Plate with Barcode, Applied Biosystems). We performed genomic qPCR using the ViiA™- 7 Real-Time PCR System (Applied Biosystems) as follows: 5 min at 95°C followed by 40 cycles [45 sec at 95°C, 30 sec

at 60°C, 45 sec at 72°C] and recorded the Ct values (cycle threshold) for each sample. We calculated the ΔCt (internal control Ct – target gene Ct) and quantified the abundance of bacteria in each sample using the 198 formula (2^ -ΔCt), normalizing the amplification of the bacteria-specific 16S rRNA gene with that of the butterfly-specific 18S rRNA gene. We performed this normalization to compare the bacterial load per unit amount of host DNA. We used previously reported primers for qPCR [13–21]. The reverse primer for host-specific 18S rRNA gene amplification was designed by Kunte lab. The relevant primer sequences are given below.

Target gene	Forward primer (5' – 3')	Reverse primer (5' – 3')
18S rRNA gene (Host specific)	CGGCTACCACATCCAAGGAA	GGCCTCGTAAGAGTCCCGTAT
16S rRNA gene (Eubacteria)	TCCTACGGGAGGCAGCAGT	GGACTACCAGGGTATCTAATCCTGTT
16S rRNA gene (Gammaproteobacteria)	CMATGCCGCGTGTGTGAA	ACTCCCCAGGCGGTCDACYTA
16S rRNA gene (Actinobacteria)	TACGGCCGCAAGGCTA	CATCCCCACCTTCCTCCG
16S rRNA gene (Firmicutes)	ACCATGCACCACCTGTC	TGAAACTYAAAGGAATTGACG

Measuring fitness-related traits of host:

- Measuring larval digestion efficiency: Along with development-related traits, we also measured larval digestion efficiency for *D. chrysippus.* We wanted to test whether larvae compensate for reduction or loss of beneficial gut bacteria by eating faster, eating more or assimilating more nutrients from the eaten leaf mass. For this, we measured the weight of the leaves given to each larva, and then measured the weight of the leftover leaf after 24 hours. We also measured the amount of the feces (frass) produced and increment in body weight for each larva during this time.

- Measuring average larvae fitness and survival across treatments: We calculated the average fitness for each measured trait in each block, and performed paired t-tests on block averages to compare fitness across treatments (see figure 3 and table S3). We report the effect sizes as Hedge's g calculated using package "effsize" in R [22–25]. Typically, effect sizes are reported as Cohen's d, which estimates standardized mean difference of an effect, calculated as "difference in the population means" / "pooled standard deviation". However, given our lower sample sizes per block ($n<20$), we used Hedge's g, which estimates Cohen's d with a correction for low sample size [23]. Whereas, we used paired t-test to compare average larval survival of all the blocks across control and treated groups.

To test the impact of bacterial elimination on the host development, we measured and compared different fitness proxies across control and treated groups. Before starting the manipulative experiments, we distributed approximately equal number of eggs in each treatment. However, all eggs in each group did not hatch. Thus, we ended up with a slightly different number of larvae in each treatment group (table S9). Also, the number of individuals in each treatment group varied slightly while measuring different fitness proxies for different developmental stages, for the following reasons. A few larvae died during the course of development and thus we could not measure their fitness at the pupal or adult stage. In rare cases, adults fell down from the pupal case right after eclosion, even before they could expand their wings. We were unsure if these adults could release meconium (metabolic waste) completely. To avoid overestimation of adult weight due to incomplete meconium release, we did not measure their body weight. In a few cases, individuals died in the pupal case and adult fitness could not be measured. None of these instances were treatment-specific. For instance, larval mortality did not vary significantly and consistently across treated and control groups across experimental blocks (Fisher's exact test, $p > 0.05$, see table S8). Finally, we also stored ~2-3 larvae from a few experimental blocks for analyzing larval bacterial communities and thus could not measure their fitness at the pupal

and adult stage. The number of individuals included in each treatment within each block is shown in table S9.

SUPPLEMENTARY TABLES

Table S1: Impact of eliminating gut bacteria on the fitness of butterfly larvae: Table shows the output of mixed-model analysis of fitness measurements. Values in bold indicate significant variation (model: fitness ~ treatment, random effect = block, $p < 0.05$). E = estimates from the model output; R^2m = proportion of variation explained by the model with only fixed effects; R^2c = proportion of variation explained by fixed and random effects together. See table S4 for all pairwise comparisons with Tukey's HSD; figures S7-S15 for block-wise analysis of fitness measurements; and table S9 for the exact number of replicates in each treatment group and fitness traits measured in each block.

	***Danaus chrysippus* Antibiotic treatment**								***Ariadne merione* Antibiotic treatment**						
**Fitness proxy**	**Compared with Sham Control (Leaves + Water)**	**E**	**R^2m**	**R^2c**	**df**	**P value**	**Lower 95% CI**	**Upper 95% CI**	**E**	**R^2m**	**R^2c**	**df**	**P value**	**Lower 95% CI**	**Upper 95% CI**
	Untreated leaves	-0.006			167	0.48	-0.02	0.01	-0.011			59	0.10	-0.02	0.01
**Adult Weight**	Leaves + Low dose antibiotic	-0.002	0.008	0.25	167	0.79	-0.02	0.02	0.003	0.06	0.53	59	0.55	-0.01	0.02
	Leaves + High dose antibiotic	-0.011			167	0.19	-0.03	0.01	-0.008			59	0.22	-0.02	0.00
	Untreated leaves	0.107			197	0.56	-0.26	0.47	-0.081			69	0.73	-0.55	0.39
**Days to eclosion**	Leaves + Low dose antibiotic	-0.097	0.004	0.27	197	0.61	-0.47	0.28	0.007	0.006	0.53	69	0.98	-0.45	0.47
	Leaves + High dose antibiotic	0.018			197	0.92	-0.34	0.38	0.144			69	0.54	-0.32	0.61
	Untreated leaves	-0.006			138	0.84	-0.06	0.05	-0.016			69	0.07	-0.03	0.00
**Pupal Weight**	Leaves + Low dose antibiotic	0.011	0.06	0.09	138	0.66	-0.04	0.06	-0.008	0.06	0.20	69	0.35	-0.02	0.01
	Leaves + High dose antibiotic	0.015			138	0.55	-0.03	0.06	-0.019			69	**0.03**	-0.04	0.00
	Untreated leaves	-0.163			194	0.42	-0.57	0.24	0.028			71	0.94	-0.66	0.71
**Days to Pupation**	Leaves + Low dose antibiotic	-0.144	0.01	0.06	194	0.49	-0.55	0.27	-0.298	0.01	0.60	71	0.38	-0.98	0.38
	Leaves + High dose antibiotic	0.089			194	0.65	-0.30	0.48	-0.443			71	0.20	-1.12	0.24
	Untreated leaves	-0.050			193	0.11	-0.11	0.01	0.050			109	**0.02**	0.01	0.09
**Larval Weight**	Leaves + Low dose antibiotic	0.012	0.01	0.46	193	0.71	-0.05	0.07	0.025	0.05	0.11	109	0.22	-0.02	0.07
	Leaves + High dose antibiotic	-0.009			193	0.77	-0.07	0.05	0.038			109	0.09	-0.01	0.08

Table S2: Impact of dietary sterilization and microbial re-introduction on the fitness of *D. chrysippus* larvae: Tables show the output of mixed-model analysis for fitness measurements. Table 2.1 shows the analysis for all 3 blocks with diet sterilization. Table 2.2 shows the analysis for 2 blocks (including microbial re-introduction).Values in bold indicate significant effects (model: fitness ~ treatment, random effect = block, *p < 0.05). E = estimates from the model output; R^2m = proportion of variation explained by the model with only fixed effects; R^2c = proportion of variation explained by fixed and random effects together. See table S9 or the exact number of replicates for each treatment group and fitness measurement for each block; and figures S13 to S15 for block-wise analysis of fitness measurements.

Table 2.1

Fitness proxy		Dietary sterilization						
	Untreated leaves compared with Sterile leaves	**E**	**R^2m**	**R^2c**	**df**	**P value**	**Lower 95% CI**	**Upper 95% CI**
**Adult Weight**		0.039	0.14	0.19	45	**0.007**	0.0014	0.098
**Days to eclosion**		-0.067	0.0003	0.73	45	0.809	-0.62	0.49
**Pupal Weight**		0.084	0.16	0.17	45	**0.005**	0.025	0.14
**Days to pupation**		-1.89	0.11	0.73	49	**<0.001**	-2.69	-1.09

Table 2.2

Fitness proxy	Compared with sham control (Sterile leaves + frass flora)	Dietary sterilization and microbial reintroduction						
		**E**	**R^2m**	**R^2c**	**df**	**P value**	**Lower 95% CI**	**Upper 95% CI**
**Adult Weight**	Sterile leaves + Leaf flora	0.01	0.16	0.16	53	0.69	-0.03	0.05
	Sterile leaves	-0.04			53	0.06	-0.08	0.00
	Untreated leaves	0.02			53	0.32	-0.02	0.06
**Days to eclosion**	Sterile leaves + Leaf flora	-0.02	0.046	0.35	50	0.63	-0.11	0.07
	Sterile leaves	-0.05			50	0.26	-0.14	0.04
	Untreated leaves	0.06			50	0.15	-0.02	0.15
**Pupal Weight**	Sterile leaves + Leaf flora	0.12	0.13	0.14	55	0.75	-0.68	0.93
	Sterile leaves	-0.40			55	0.31	-1.19	0.39
	Untreated leaves	-0.55			55	0.18	-1.37	0.26
**Days to pupation**	Sterile leaves + Leaf flora	-0.70	0.20	0.22	60	0.26	-1.94	0.53
	Sterile leaves	-0.08			60	0.89	-1.29	1.13
	Untreated leaves	-2.15			60	**0.001**	-3.40	-0.90

Table S3: A summary of fitness-related traits across control and treated groups. Table shows the mean of block averages (i.e. mean of means), with associated standard deviation (SD). The effect size (Hedge's "g") indicates the magnitude of the difference between the treated vs. control group, with the associated p value derived from paired t-tests. Raw data and statistical analysis for individual experimental blocks is shown in figures S7-S15 and tables S5-S7. See table S9 for the exact number of replicates for each treatment group and fitness measurement.

**Antibiotic treatment – *Danaus chrysippus***															
**Fitness proxy**	**blocks**	**Leaves + Water**		**Leaves + low dose antibiotic**						**Leaves + high dose antibiotic**					
		Mean	SD	Mean	SD	Effect Size	Lower 95% CI	Upper 95% CI	p value	Mean	SD	Effect Size	Lower 95% CI	Upper 95% CI	p value
Larval Weight	3	**0.49**	0.15	**0.51**	0.20	-0.10	-2.37	2.16	0.52	**0.50**	0.18	-0.03	-2.29	2.23	0.72
Days to Pupation	4	**13.53**	0.45	**13.51**	0.77	0.013	-1.71	1.74	0.96	**13.74**	0.45	-0.44	-2.19	1.30	0.07
Pupal Weight	3	**0.59**	0.01	**0.60**	0.04	-0.18	-2.45	2.08	0.81	**0.59**	0.04	-0.09	-2.36	2.17	0.89
Days to Eclosion	4	**10.03**	0.30	**10.02**	0.59	0.01	-1.72	1.73	0.97	**10.21**	1.06	-0.19	-1.93	1.53	0.69
Adult Weight	4	**0.26**	0.03	**0.25**	0.02	0.14	-1.58	1.87	0.55	**0.24**	0.03	0.53	-1.22	2.29	0.26
Larval Survival	4	**97**	3	**90**	12	0.69	-1.08	2.47	0.37	**99**	2	-0.51	-2.26	1.24	0.40
**Antibiotic treatment – *Ariadne merione***															
Larval Weight	3	**0.14**	0.059	**0.14**	0.057	-0.01	-2.28	2.24	0.95	**0.14**	0.074	-0.03	-2.30	2.22	0.96
Days to Pupation	2	**17.2**	2.10	**16.7**	1.20	0.14	-4.15	4.45	0.60	**16.6**	1.07	0.22	-4.09	4.53	0.55
Pupal Weight	2	**0.28**	0.03	**0.27**	0.002	0.34	-3.99	4.67	0.68	**0.26**	0.01	0.53	-3.84	4.90	0.36
Days to Eclosion	2	**8.27**	0.80	**8.29**	0.41	-0.04	-4.34	4.25	0.94	**8.36**	0.90	-0.06	-4.37	4.23	0.39
Adult Weight	2	**0.12**	0.01	**0.12**	0.004	-0.25	-4.57	4.06	0.96	**0.11**	0.01	0.76	-3.69	5.22	0.06
Larval Survival	3	**80**	7	**80**	18	-0.06	-2.32	2.20	0.98	**67**	32	0.43	-1.85	2.72	0.62

**Dietary sterilization – *Danaus chrysippus***													
		**Untreated leaves**		**Sterile leaves + Feces flora**		**Sterile leaves + Leaf flora**		**Sterile leaves (compared with untreated control)**					
		Mean	SD	Mean	SD	Mean	SD	Mean	SD	Effect Size	Lower 95% CI	Upper 95% CI	p value
Days to Pupation	3	**13.17**	2.74	**17.0**	0.87	**16.1**	1.49	**14.99**	3.36	-0.47	-2.77	1.82	0.08
Pupal Weight	3	**0.637**	0.048	**0.619**	0.0004	**0.583**	0.05	**0.561**	0.03	1.52	-1.05	4.1	0.10
Days to Eclosion	3	**10.85**	1.45	**12.25**	0.35	**12.37**	0.68	**10.99**	1.87	-0.07	-2.33	2.19	0.73
Adult Weight	3	**0.284**	0.030	**0.281**	0.013	**0.289**	0.0054	**0.244**	0.02	1.35	-1.15	3.87	0.16
Larval Survival	3	**94**	11	**80**	3.5	**100**	0	**90**	12	0.28	-1.99	2.56	0.2

Table S4: An output of Tukey's HSD performed on mixed models to compare larval fitness across treatments. Table S4.1 and S4.2 show the pairwise comparisons across treatments using Tukey's HSD test performed on a mixed model. Table S1 summarizes the output of the mixed model analysis. See table S9 for the exact number of replicates for each treatment group and fitness measurement.

**Table S4.1**		***Danaus chrysippus* (Antibiotic treatment)**				***Ariadne merione* (Antibiotic treatment)**			
Fitness proxy	Pairwise comparisons	Estimate	p Value	Lower 95% CI	Upper 95% CI	Estimate	p Value	Lower 95% CI	Upper 95% CI
Larval Weight	UT-LW	-0.050	0.38	-0.131	0.030	0.050	0.07	-0.002	0.102
	LD-LW	0.012	0.98	-0.070	0.094	0.025	0.61	-0.028	0.079
	HD-LW	-0.009	0.99	-0.087	0.069	0.038	0.30	-0.018	0.094
	LD-UT	0.062	0.22	-0.021	0.145	-0.024	0.63	-0.077	0.028
	HD-LW	0.041	0.55	-0.039	0.121	-0.012	0.95	-0.067	0.044
	HD-LD	-0.021	0.91	-0.102	0.060	0.012	0.94	-0.044	0.068
Days to pupation	UT-LW	-0.163	0.85	-0.687	0.360	0.028	1.00	-0.856	0.911
	LD-LW	-0.144	0.90	-0.677	0.390	-0.298	0.82	-1.170	0.573
	HD-LW	0.089	0.97	-0.418	0.596	-0.443	0.56	-1.317	0.431
	LD-UT	0.020	1.00	-0.517	0.556	-0.326	0.75	-1.165	0.512
	HD-LW	0.252	0.58	-0.258	0.763	-0.471	0.47	-1.309	0.366
	HD-LD	0.233	0.66	-0.286	0.751	-0.145	0.97	-0.962	0.671
Pupal Weight	UT-LW	-0.006	1.00	-0.079	0.068	-0.016	0.24	-0.038	0.006
	LD-LW	0.011	0.97	-0.054	0.076	-0.008	0.79	-0.029	0.014
	HD-LW	0.015	0.93	-0.050	0.080	-0.019	0.12	-0.040	0.003
	LD-UT	0.017	0.93	-0.054	0.087	0.008	0.75	-0.013	0.029
	HD-LW	0.021	0.87	-0.049	0.091	-0.003	0.99	-0.024	0.018
	HD-LD	0.004	1.00	-0.057	0.065	-0.011	0.52	-0.031	0.010
Days to eclosion	UT-LW	0.184	0.94	-0.365	0.579	-0.081	0.99	-0.688	0.527
	LD-LW	0.190	0.96	-0.586	0.391	0.007	1.00	-0.584	0.598
	HD-LW	0.182	1.00	-0.449	0.485	0.144	0.93	-0.455	0.742
	LD-UT	0.190	0.70	-0.694	0.284	0.088	0.98	-0.492	0.668
	HD-LW	0.183	0.96	-0.558	0.380	0.225	0.76	-0.359	0.808
	HD-LD	0.188	0.93	-0.367	0.598	0.136	0.92	-0.426	0.699
Adult Weight	UT-LW	-0.006	0.89	-0.026	0.015	-0.012	0.24	-0.029	0.005
	LD-LW	-0.002	0.99	-0.026	0.021	0.003	0.96	-0.013	0.019
	HD-LW	-0.011	0.55	-0.031	0.010	-0.011	0.28	-0.027	0.005
	LD-UT	0.003	0.98	-0.020	0.026	0.015	0.06	0.000	0.031
	HD-LW	-0.005	0.93	-0.025	0.016	0.001	1.00	-0.015	0.017
	HD-LD	-0.008	0.79	-0.031	0.015	-0.014	0.07	-0.029	0.001

**Table S4.2**		***Danaus chrysippus*** (Dietary sterilization and microbial reintroduction)			
Fitness proxy	Pairwise comparisons	Estimate	p Value	Lower 95% CI	Upper 95% CI
Larval Weight	LF-FF	-0.70	0.67	-2.29	0.88
	SL-FF	-0.08	1.00	-1.63	1.47
	UT-FF	-2.15	0.003	-3.76	-0.54
	SL-LF	0.62	0.68	-0.82	2.06
	UT-LF	-1.45	0.06	-2.95	0.06
	UT-SL	-2.07	0.001	-3.53	-0.61
Days to pupation	LF-FF	-0.02	0.96	-0.13	0.09
	SL-FF	-0.05	0.66	-0.16	0.06
	UT-FF	0.06	0.47	-0.05	0.17
	SL-LF	-0.03	0.90	-0.14	0.08
	UT-LF	0.08	0.18	-0.02	0.19
	UT-SL	0.11	0.03	0.01	0.22
Pupal Weight	LF-FF	0.12	0.99	-0.90	1.15
	SL-FF	-0.40	0.74	-1.41	0.62
	UT-FF	-0.55	0.52	-1.60	0.49
	SL-LF	-0.52	0.48	-1.46	0.41
	UT-LF	-0.68	0.28	-1.64	0.29
	UT-SL	-0.15	0.98	-1.11	0.80
Days to eclosion	LF-FF	0.01	0.98	-0.05	0.06
	SL-FF	-0.04	0.22	-0.09	0.01
	UT-FF	0.02	0.75	-0.03	0.07
	SL-LF	-0.05	0.07	-0.10	0.00
	UT-LF	0.01	0.93	-0.04	0.06
	UT-SL	0.06	0.01	0.01	0.11

Table S5: Impact of antibiotic treatment on the fitness-related traits of *D. chrysippus.* Table 5.1 shows the treatments included in each experimental block. Table 5.2 shows the results of analyses of fitness measurements across treatments (fitness of antibiotic treated groups is compared with group B; generalized linear model, Tukey's post- hoc test for multiple comparisons). Asterisks indicate significant variation (*p<0.05). "E" represents the "estimate" from the Tukey's post hoc test and is reported only for the comparisons that are significant. Replicate size per experimental block is represented as a range (n per block). Non-significant comparisons are indicated as "ns", and fitness proxies that were not determined are indicated by "nd". See table S9 for the exact number of replicates for each treatment group and fitness measurement.

5.1: Treatments included in each experimental block

	**A**	**B**	**C**	**D**
Treatments	**Untreated leaves**	**Leaves + Water**	**Leaves + Low Dose antibiotic**	**Leaves + High Dose antibiotic**
**Block 1**	✓	✓	✓	✓
**Block 2**	✓	✓	✓	✓
**Block 3**	✓	✓	✓	✓
**Block 4**	✓	✓	✓	✓

5.2: Impact of the antibiotic treatment on *D. chrysippus* fitness

**Experimental blocks**	**n per block**	**Larval Length**	**Larval Weight**	**Days to pupation**	**Pupal Weight**	**Days to eclosion**	**Adult Weight**	**Larval digestion efficiency**
Block 1	5-8	ns	ns	ns	ns	ns	ns	nd
Block 2	4-18	ns	ns	ns	ns	ns	ns	*B>C p= 0.04, E= -0.15<br>Weight gained per gram of leaf eaten by 11-day old larvae.
Block 3	9-25	ns	ns	ns	ns	ns	ns	ns
Block 4	4-13	ns	ns	ns	ns	ns	ns	ns

Table S6: Impact of antibiotic treatment on fitness-related traits of *A. merione*. Table 6.1 shows treatments included in each experimental block. Table 6.2 shows the results of analyses of fitness measurements across treatments (fitness of antibiotic treated groups is compared with group B, generalized linear model, Tukey's post- hoc test for multiple comparisons). Asterisks indicate significant variation (*p<0.05). "E" represents the "estimate" from the Tukey's post-hoc test and is reported only for the comparisons that are significant. Non-significant comparisons are indicated as "ns", and fitness proxies that were not determined are indicated by "nd". See table S9 for the exact number of replicates for each treatment group and fitness measurement.

6.1: Treatments per block

Treatments	**A**<br>**Untreated leaves**	**B**<br>**Leaves + Water**	**C**<br>**Leaves + Low Dose antibiotic**	**D**<br>**Leaves + High Dose antibiotic**
**Block 1**	✓	✓	✓	✓
**Block 2**	✓	✓	✓	✓
**Block 3**	✓	✓	✓	✓

6.2: Impact of the antibiotic treatment on *A. merione* fitness

**Experimental blocks**	**n per block**	**Larval Length**	**Larval Weight**	**Days to pupation**	**Pupal Weight**	**Days to eclosion**	**Adult Weight**
Block 1	9-16	ns	ns	ns	ns	ns	ns
Block 2	5-12	ns	ns	ns	B>D* ,p=0.005, E= -0.04<br>B>C* ,p=0.02, E= -0.03<br>B>A* ,p=0.01, E= 0.04	ns	B>A* p=0.01, E= 32
Block 3	3-8	B>D*, p=0.04, E= -4.1	ns	nd	nd	nd	nd

Table S7: Impact of dietary sterilization on fitness-related traits of *D. chrysippus*. Table 7.1 shows the treatments included in each experimental block. Table 7.2 shows the results of analyses of fitness measurements across treatments. Asterisks indicate significant variation between the control group (treatment A) and other treatments (Generalized linear model, Tukey's post hoc test for multiple comparisons, *$p < 0.05$), with the direction of the difference as indicated. "E" represents the "estimate" from the Tukey's post-hoc test and is reported only for the comparisons that are significant. Non-significant comparisons are indicated as "ns", and fitness proxies that were not determined are indicated by "nd". See table S9 for the exact number of replicates for each treatment group and fitness measurement.

7.1: Treatments included in each experimental block

Treatments	A	B	C	D
	**Unsterile Diet**	**Sterile Diet + Frass flora**	**Sterile Diet + Leaf flora**	**Sterile Diet**
**Block 1**	✓	nd	nd	✓
**Block 2**	✓	✓	✓	✓
**Block 3**	✓	✓	✓	✓

7.2: Impact of dietary sterilization and microbial reintroduction on *D. chrysippus* fitness

Experimental blocks	n per block	Larval Length	Larval Weight	Days to pupation	Pupal Weight	Days to eclosion	Adult Weight
**Block 1**	8-9	*D<A, p= 9.44e-05, E = -9.1	*D<A, p=0.0231,E = -0.10	*D>A, p=0.025,E= 11.2	ns	ns	ns
**Block 2**	6-13	*D<A, p=0.04, E= 3.1	nd	ns	ns	ns	*D<A, p=0.04E = -0.06
**Block 3**	6-8	ns	nd	*B>A, p=0.001, E=3.6<br>*D>A, p=0.01, E= 2.8	ns	ns	ns

Table S8: Larval mortality across experimental blocks and treatment. We did not observe significant difference in mortality across treatments (tested separately for each block - across control and treatment groups in a pairwise manner, Fisher's exact test, $p>0.05$ in each case, except in *A. merione* block 3: $p=0.02$*, mortality in (Leaves + Water) > (Leaves + High Dose antibiotic).

**Dietary sterilization**	**Unsterile leaves**		**Sterile leaves**		**Sterile leaves + leaf flora**		**Sterile leaves+ frass flora**		**Fisher's exact test**
	Total number of larvae	% Dead larvae	Total number of larvae	% Dead larvae	Total number of larvae	% Dead larvae	Total number of larvae	% Dead larvae	p Value
D. chrysippus block 1	9	0%	9	0%	--	--	--	--	No Mortality
D. chrysippus block 2	13	0%	15	7%	12	0%	13	23%	p>0.05
D. chrysippus block 3	11	18%	13	23%	11	0%	11	18%	p>0.05
**Antibiotic treatment**	**Untreated leaves**		**Leaves + Water**		**Leaves + Low dose antibiotics**		**Leaves + High dose antibiotics**		
D. chrysippus block 1	5	0%	5	0%	8	0%	8	0%	No Mortality
D. chrysippus block 2	14	7%	16	6%	15	7%	18	0%	p>0.05
D. chrysippus block 3	29	10%	26	4%	23	4%	25	4%	p>0.05
D. chrysippus block 4	13	0%	13	0%	11	27%	13	0%	p>0.05
A.merione block 1	18	6%	17	24%	19	26%	19	21%	p>0.05
A.merione block 2	11	36%	8	25%	12	0%	11	9%	p>0.05
A.merione block 3	10	20%	8	13%	9	33%	10	70%	P<0.05*

Table S9: Replicate sizes across different experimental blocks. Tables 9.1, 9.2 and 9.3 show the number of larvae tested in each experimental block. Fitness proxies that were not determined are represented as "nd". See tables S5-S7 for a description of treatments.

9.1 Testing the impact of dietary sterilization on the fitness of *D. chrysippus*

	Block 1		Block 2				Block 3			
Treatments	**A**	**D**	**A**	**B**	**C**	**D**	**A**	**B**	**C**	**D**
Number of larvae at the beginning of the experiment	9	9	13	13	12	15	11	11	11	13
Larval Length	9	9	13	13	12	15	10	11	11	12
Larval Weight	9	9	nd				nd			
Days to pupation	8	9	10	7	9	12	6	6	8	7
Pupal Weight	9	9	10	7	7	7	6	5	7	5
Days to eclosion	9	9	9	6	9	10	6	6	8	7
Adult Weight	9	8	10	6	7	7	6	6	7	7

9.2 Testing the impact of antibiotic treatment on the fitness of *A. merione*

	Block 1				Block 2				Block 3			
Treatments	**A**	**B**	**C**	**D**	**A**	**B**	**C**	**D**	**A**	**B**	**C**	**D**
Number of larvae at the beginning of the experiment	18	17	18	20	11	6	12	11	8	8	9	10
Larval Length	16	14	14	14	7	6	12	11	8	7	6	3
Larval Weight	16	13	12	13	8	6	12	8	8	7	6	3
Days to pupation	13	10	9	11	6	6	12	9	nd			
Pupal Weight	13	10	9	11	5	6	11	9	nd			
Days to eclosion	13	10	10	11	5	6	12	9	nd			
Adult Weight	13	9	9	11	5	6	12	9	nd			

9.3 Testing the impact of antibiotic treatment on the fitness of *D. chrysippus*

	Block 1				Block 2				Block 3				Block 4			
Treatments	**A**	**B**	**C**	**D**	**A**	**B**	**C**	**D**	**A**	**B**	**C**	**D**	**A**	**B**	**C**	**D**
Number of larvae at the beginning of the experiment	5	5	8	8	14	16	15	18	29	26	23	25	13	13	11	13
Larval Length	5	5	8	8	13	15	14	18	24	25	16	19	13	13	8	12
Larval Weight	5	5	8	8	9	15	14	16	19	17	15	18	13	13	7	12
Days to pupation	5	5	8	8	12	15	14	18	23	17	16	15	13	13	8	12
Larval digestion efficiency	nd				8	13	14	15	20	14	15	15	8	6	4	8
Pupal Weight	nd				6	8	11	9	22	19	20	21	11	11	8	11
Days to eclosion	5	5	8	8	12	15	14	18	22	19	19	19	13	13	6	10
Adult Weight	5	5	8	8	12	14	12	18	20	15	9	13	11	12	4	9

REFERENCES:

1. Brummel T, Ching A, Seroude L, Simon AF, Benzer S. 2004 *Drosophila* lifespan enhancement by exogenous bacteria. *Proc. Natl. Acad. Sci.* **101**, 12974–9. (doi:10.1073/pnas.0405207101)
2. Gil Sharon, Daniel Segal, John M. Ringo, Abraham Hefetz, Ilana Zilber-Rosenberg and ER. 2013 Correction for Sharon et al., Commensal bacteria play a role in mating preference of *Drosophila melanogaster*. *Proc. Natl. Acad. Sci.* **110**, 4853 LP – 4853. (doi:10.1073/pnas.1302326110)
3. Gaio A de O, Gusmão DS, Santos A V, Berbert-Molina MA, Pimenta PFP, Lemos FJA. 2011 Contribution of midgut bacteria to blood digestion and egg production in *Aedes aegypti* (diptera: culicidae) (L.). *Parasit. Vectors* **4**, 105. (doi:10.1186/1756-3305-4-105)
4. Salter SJ *et al.* 2014 Reagent and laboratory contamination can critically impact sequence-based microbiome analyses. *BMC Biol.* **12**, 87. (doi:10.1186/s12915-014-0087-z)
5. Phalnikar K, Kunte K, Agashe D. 2018 Dietary and developmental shifts in butterfly-associated bacterial communities. *R. Soc. open Sci.* **5**, 171559. (doi:10.1098/rsos.171559)
6. Illumina. 2013 16s metagenomic sequencing library preparation (https://support.illumina.com/documents/documentation/chemistry_documentation/16s/16s-metagenomic-library-prep-guide-15044223-b.pdf).
7. Caporaso JG *et al.* 2010 QIIME allows analysis of high-throughput community sequencing data. *Nat. Methods* **7**, 335–336. (doi:10.1038/nmeth.f.303)
8. Edgar RC. 2010 Search and clustering orders of magnitude faster than BLAST. *Bioinformatics* **26**, 2460–2461. (doi:10.1093/bioinformatics/btq461)
9. Oksanen J *et al.* 2017 vegan: Community Ecology Package. *R Packag. ver. 2.4–3* (doi:10.4135/9781412971874.n145)
10. Team RC. 2013 R: A language and environment for statistical computing.
11. Weiner J. 2015 pca3d: Three dimensional PCA plots. *R Packag. version 0.8* **484**.
12. Kindt R. 2016 BiodiversityR: package for community ecology and suitability analysis. *R version* **2.7**.
13. Bacchetti T, Gregoris D, Aldred N, Clare AS, Burgess JG. 2011 Improvement of phylum- and class-specific primers for real-time PCR quantification of bacterial taxa. **86**, 351–356. (doi:10.1016/j.mimet.2011.06.010)
14. Westfall S, Lomis N, Prakash S. 2018 A novel polyphenolic prebiotic and probiotic formulation have synergistic effects on the gut microbiota influencing *Drosophila melanogaster* physiology. *Artif. Cells, Nanomedicine, Biotechnol.* **0**, 1–15. (doi:10.1080/21691401.2018.1458731)
15. Pedersen R, Andersen AD, Mølbak L, Stagsted J, Boye M. 2013 Changes in the gut microbiota of cloned and non-cloned control pigs during development of obesity : gut microbiota during development of obesity in cloned pigs. , 1–9. (doi:10.1186/1471-2180-13-30)
16. Choi S, Park HY, Paek A, Kim GS, Jeong SE. 2009 Insect Ornithine Decarboxylase (ODC) complements SPE1 knock-out of yeast *Saccharomyces cerevisiae*. , 575–581. (doi:10.1007/s10059-009-0162-4)
17. Liang P, Guo Y, Zhou X, Gao X. 2014 Expression profiling in *Bemisia tabaci* under insecticide treatment : Indicating the necessity for custom reference gene selection.
18. Mohammadi T, Reesink HW, Vandenbroucke-Grauls CMJE, Savelkoul PHM. 2003 Optimization of real-time PCR assay for rapid and sensitive detection of eubacterial 16S ribosomal DNA in platelet concentrates. *J. Clin. Microbiol.* **41**, 4796–4798. (doi:10.1128/jcm.41.10.4796-4798.2003)
19. Zucol F, Ammann RA, Berger C, Aebi C, Altwegg M, Niggli FK, Nadal D. 2006 Real-time quantitative broad-range PCR assay for detection of the 16S rRNA gene followed by sequencing for species identification. *J. Clin. Microbiol.* **44**, 2750 LP – 2759. (doi:10.1128/JCM.00112-06)
20. Tarpy DR, Mattila HR, Newton ILG. 2015 Development of the honey bee gut microbiome throughout the queen-rearing process. *Appl. Environ. Microbiol.* **81**, 3182 LP – 3191. (doi:10.1128/AEM.00307-15)

21. Wei G, Lai Y, Wang G, Chen H, Li F, Wang S. 2017 Insect pathogenic fungus interacts with the gut microbiota to accelerate mosquito mortality. *Proc. Natl. Acad. Sci.* **114**, 5994 LP – 5999. (doi:10.1073/pnas.1703546114)
22. Turner HM, Bernard RM. 2006 Calculating and synthesizing effect sizes. *Contemp. issues Commun. Sci. Disord.* **33**, 42–55. (doi:10.1044/cicsd_33_S_42)
23. Torchiano M, Torchiano MM. 2019 Package 'effsize'.
24. Lakens D. 2013 Calculating and reporting effect sizes to facilitate cumulative science: a practical primer for t-tests and ANOVAs. *Front. Psychol.* **4**, 863. (doi:doi:10.3389/fpsyg.2013.00863)
25. Cohen J. 2013 *Statistical power analysis for the behavioral sciences*. Routledge.
